# When ChatGPT Writes Your Research Proposal: Scientific Creativity in the Age of Generative AI

**DOI:** 10.3390/jintelligence13050055

**Published:** 2025-05-16

**Authors:** Vera Eymann, Thomas Lachmann, Daniela Czernochowski

**Affiliations:** 1 Center for Cognitive Science, University of Kaiserslautern-Landau (RPTU), 67663 Kaiserslautern, Germany; lachmann@rptu.de (T.L.); d.czernochowski@rptu.de (D.C.); 2 Centro de Investigación Nebrija en Cognición (CINC), Universidad Nebrija, 28015 Madrid, Spain; 3 Brain and Cognition Research Unit, Faculty of Psychology and Educational Sciences, KU Leuven, 3000 Leuven, Belgium

**Keywords:** artificial intelligence, scientific creativity, creativity, intelligence, ChatGPT

## Abstract

Within the last years, generative artificial intelligence (AI) has not only entered the field of creativity; it might even be marking a turning point for some creative domains. This raises the question of whether AI also poses a turning point for scientific creativity, which comprises the ability to develop new ideas or methodological approaches in science. In this study, we use a new scientific creativity task to investigate the extent to which AI—in this case, ChatGPT-4—can generate creative ideas in a scientific context. Specifically, we compare AI-generated responses with those of graduate students in terms of their ability to generate scientific hypotheses, design experiments, and justify their ideas for a fictitious research scenario in the field of experimental psychology. We asked students to write and prompted ChatGPT to generate a brief version of a research proposal containing four separate assignments (i.e., formulating a hypothesis, designing an experiment, listing the required equipment, and justifying the chosen method). Using a structured (blinded) rating, two experts from the field evaluated students’ research proposals and proposals generated by ChatGPT in terms of their scientific creativity. Our results indicate that ChatGPT received significantly higher overall scores, but even more crucially exceeded students in sub-scores measuring originality or meaningfulness of the ideas. In addition to a statistical evaluation, we qualitatively assess our data providing a more detailed report in regards to subtle differences between students’ and AI-generated responses. Lastly, we discuss challenges and provide potential future directions for the field.

## 1. Introduction

Creativity is defined as the ability to produce a novel but also appropriate idea or work (Lubart 1994; Runco and Jaeger 2012). With this definition, proper creativity has to meet two requirements: (1) to be original and uncommon, rather than just a simple adaptation or minor improvement and (2) to be adaptive in the sense of matching the respective situation in order to respond to a problem. More recently, creativity was additionally characterized by intentionality and authenticity (see (Runco, 2023) for details) as well as embodied cognition and emotional depth (Lockhart 2024), thus emphasizing the complexities of *human* creativity. In standardized creativity tasks, ideas are usually evaluated in terms of fluency (i.e., the number of produced ideas), flexibility (i.e., number of categories of ideas), and originality (i.e., how statistically rare an idea is (Runco and Acar 2012)). Furthermore, creativity and human intelligence are regarded as very closely intertwined, although there is no final consensus on their relationship yet (see (Sternberg and O’Hara 2000) as well as (Kaufman and Plucker 2011) for overviews). And with that, it seems to be rather unlikely to classify artificial intelligence (AI) as creative (Vinchon et al. 2024) and able to generate truly novel ideas and innovative solutions (Runco and Jaeger 2012) beyond the scope of their training data (Farina et al. 2024; Opara 2025).

Within the last few years, we experience a surge of Generative AIs which generate verbal or even figural content from existing information. Probably the most famous one is ChatGPT developed by OpenAI. ChatGPT in the current version of ChatGPT-4 is designed as a chatbot that is based on the large language model (LLM) GPT (Generative Pre-trained Transformer). It can understand complex commands (so called prompts) and natural language queries from which it generates coherent responses and fluid, natural conversations (Liu 2024) even for complex scenarios (OpenAI 2023). Due to its architecture, ChatGPT will generate its output by “seeking to statistically predict which words come one after the other according to the given input” (Vinchon et al. 2024, p. 2).

While at first glance, this approach does not seem strikingly creative and does not meet the full definition of Runco (2023), generative AIs have already entered several artistic/creative domains, such as painting art, music creation, poetry, story writing, and movie scripting (see (Vinchon et al. 2024) as well as (Formosa et al. 2024) for comprehensive overviews). In addition, some recent studies suggest that LLMs such as ChatGPT already performed similarly to or even exceeded human norms. For example, Guzik et al. (2023) reported that ChatGPT achieved excellent scores for the verbal Torrance Tests of Creative Thinking (TTCT; Lissitz and Willhoft 1985), especially with respect to idea fluency and flexibility, but more critically also for original thinking. On the other hand, in specific activities to assess flexibility in the TTCT, such as “guessing causes” or “guessing consequences” (Guzik et al. 2023), ChatGPT scored relatively lower while still outperforming the human control group (undergraduate students). The authors concluded that this might be caused by the type of prompts provided to ChatGPT to test these categories (Guzik et al. 2023). In the study of Orwig et al. (2024), short stories generated by ChatGPT obtained creativity scores comparable to the human sample. Lastly, Vinchon et al. (2024) reported that ChatGPT performed particularly well in terms of fluency, but at the same time it noticeably plagiarized from well-known stories in a creative production of stories task of the EPoC-test (Evaluation of Potential Creativity; Barbot et al. 2016). Thus, in standardized tests as well as when assessed by human raters (e.g., Si et al. 2024), AI-generated solutions match or even outperform humans. However, this does not imply that AI follows the same (cognitive) operations as humans to generate novel ideas; it merely illustrates that AI mimics human creativity.

In addition, ChatGPT-4 does not only surpass other generative AI applications, it also exhibits human-level performance in various academic exams and demonstrates proficiency across several academic disciplines, such as biology, mathematics, and history, even without specific training for these particular exams (OpenAI 2023). This means ChatGPT-4 is capable of working with the same material as humans to take an exam on an academic level and hence understands the knowledge base as a prerequisite for scientific research. Scientific discovery, its automatization, and even the implementation of the “AI scientist” (see (Kitano 2021) for overview) have been major topics in AI research for many years. Well-known examples include AM and EURISKO (Lenat and Brown 1984), as well as BEACON (Langley 1987). However, with the public availability of generative AI applications such as ChatGPT, their relevance for the scientific community has skyrocketed. In this regard, ChatGPT entered and was tested in different scientific processes such as generation of research ideas (e.g., (Si et al. 2024; Girotra et al. 2023)), hypotheses generation (e.g., (Ghafarollahi and Buehler 2024)), literature search (e.g., (Ghafarollahi and Buehler 2024)), or even writing empirical research papers (e.g., (Wang et al. 2019)) or scientific reviews (e.g., (Huang and Tan 2023)). There are already published articles that list ChatGPT as one of the authors, indicating that ChatGPT has made a significant contribution to the content of the manuscript (Formosa et al. 2024). This leads to the question whether AI might be able to actually make scientific contributions such as (groundbreaking) scientific discoveries. In other words: Does AI have the potential to co-work alongside or even to replace scientists in these tasks?

### 1.1. Scientific Creativity

Scientific creativity can be described as the ability to develop new ideas or approaches in the scientific field. It also refers to the “ability of conducting creative science experiments and finding out and solving creative science problems and science activities” (Raj and Saxena 2016, p. 1122). Kramer et al. (2023) formalized the scientific process into six possibly recursive steps: (1) Formulating a scientific question (describing the phenomenon), (2) Formulating a hypothesis, (3) Designing an experiment to test the hypotheses, (4) Performing the experiment, (5) Analyzing the experimental results, and (6) Communication of results to the scientific community (see (Kramer et al. 2023) for details). Within this framework, the initial three steps in particular rely on creativity, but fundamentally also on domain-specific knowledge (Beaty et al. 2024). Hence, it is necessary to test scientific creativity in a framework specifically designed for each of the respective scientific fields.

Lubart et al. (2022) concluded that there are three main ways to detect creative potential in science: Accomplishment-based measures (e.g., number of impactful publications), science-based competitions (e.g., science talent competitions), and psychometric testing (i.e., producing scientific ideas for a given problem). However, while scientific creativity has been investigated in various ways (see (Raj and Saxena 2016) for a comprehensive overview), as of today, most of the accessible tests on scientific creativity are targeted towards (high) school students. These tests oftentimes neither require domain-specific knowledge, nor are necessarily appropriate for scientific environments, as they are more suited for a school setting, testing children and adolescents (Lubart et al. 2022). There are a few domain-specific tests (such as (Beaty et al. 2024) or (Sternberg and Sternberg 2017)), which specifically target university students from STEM (Science, Technology, Engineering, and Mathematics; (Beaty et al. 2024)) or humanities (e.g., psychology or education; (Sternberg and Sternberg 2017)). In the latter, students are asked to formulate hypotheses or design experiments for several field-related examples. The authors further concluded that these types of assessment could be of value in order to assess the potential success of graduate students in their aspired career as researchers as opposed to traditional academic exams.

To summarize, scientific creativity requires creative thinking as well as domain-specific knowledge (Beaty et al. 2024). Hence, it is appropriate to assess scientific creativity in a framework specifically designed for the respective scientific field. Because the scientific process is carried out in multiple stages (Kramer et al. 2023), these different activities can be separated and operationalized in a task to assess scientific creativity in a way that resembles the actual work of a scientist (such as writing grant proposals). As it has been shown that ChatGPT can process input on an academic level and has previously managed to succeed in different stages of the scientific process (e.g., generation of research ideas), it is reasonable to test its capabilities in this particular way.

### 1.2. The Present Study

In this study, we investigate whether AI-generated contributions with respect to an abbreviated research proposal containing four specific assignments (i.e., formulating a hypothesis, designing an experiment, listing the required equipment, and justifying the chosen method) can mimic or outperform graduate students. Following the implications of the study of Sternberg and Sternberg (2017), we aimed to design the task very similar to the actual work of a scientist. Since AI vastly outperforms humans in the speed and amount of text production (fluency), we limited the responses to a very narrow scenario in a specific research topic. Humans and AI received the same natural language instructions for a fictitious research scenario. We also limited the structure of the proposal by specifying the desired length and, hence, how many details could be mentioned (see below).

## 2. Materials and Methods

The human sample consisted of 10 graduate students (5 female; age range from 22 to 27; 1 student was a native English speaker) currently enrolled in the Cognitive Science study program at the University of Kaiserslautern-Landau. This interdisciplinary study program is research-focused and most graduate students aspire to a career in research after completing the program. To our knowledge, this is the first scientific creativity task that is specifically targeted to the field of cognitive science, taking into account the interdisciplinary nature of this field. Hence, our task involves four different scenarios for the respective sub-disciplines of neuroscience, linguistics, computer science, and psychology (Eymann et al. 2024a). To assess the creative potential of ChatGPT, we used the psychology scenario of the task (see below for details). Human participants were asked to generate a brief version of a research proposal to assess a fictitious scenario describing one to-be-challenged explanation as well as one alternative approach to explain a phenomenon which may be the topic of an empirical investigation. The human data were collected as part of a larger study on scientific creativity. Students were placed in a quiet room in groups of up to 10 participants to answer four structured questions in written format. There was no time restriction, but participants were given instructions for how long the answers were expected to be (see details for each assignment below). This was performed to avoid an extensive word count for AI-generated responses and at the same time ensure comparability between all participants. Students were compensated for their participation with course credits. The AI submissions were generated by prompting 5 different OpenAI ChatGPT-4 applications in the Department for Cognitive and Developmental Psychology at the University of Kaiserslautern-Landau. The temperature of all ChatGPT-4 applications was set uniformly at 1.0, corresponding to a high value which enhances variability and randomness in the generated responses (Davis et al. 2024); this also corresponds to the default value of ChatGPT-4 (OpenAI 2023) and hence reflects a high ecological validity.

Our research utilized a recently developed scientific creativity task specifically designed to assess Master students from the Cognitive Science study program of the University of Kaiserslautern-Landau. In this task, we introduced students and prompted ChatGPT-4 to the following fictious scenario from the field of experimental psychology:

“*A recent survey showed that university students in Rhineland-Palatinate who are introverts tend to order pizza with peppers and mushrooms, while university students that rate themselves as extroverts tend to order pizza with eggplant and corn. At the same time, these introverted students also stated that they like rock music, while the extroverts stated that they like to listen to chill out music. Imagine you are an experienced scientist working at the Department of Psychology at the University of Kaiserslautern-Landau. Your colleague argues that this must have something to do with their diet, because eggplant contains a high amount of magnesium which makes the extrovert students more relaxed and thus more interested in relaxing music. You are skeptical because you think that the preference for music has more to do with a personality trait rather than a person’s diet. But how could you test this? Please outline a short research proposal to convince a jury of the German Research Foundation to fund your research.*”

Students were then asked to work on the following four different assignments for this scenario:

### 2.1. Assignment 1—Generating a Hypothesis

Participants were asked to write down the specific hypothesis that they would want to test in 1–2 sentences. This assignment was used to ensure that participants correctly understood the scenario and to understand their thought process regarding the experimental procedure. It was further important that the hypothesis they formulated was testable and logical.

### 2.2. Assignment 2—Outlining the Procedure

Participants were asked to outline the exact procedure of their proposed experiment using 3–5 sentences. Here, it was important that participants produced a procedure with the corresponding operationalizations that were logical, valid, and feasible.

### 2.3. Assignment 3—Listing Necessary Equipment

In this assignment, participants were asked to list the equipment they would need to conduct their proposed experiment using bullet points.

### 2.4. Assignment 4—Reasoning of Rationale

This last assignment asked participants to explain their rationale, i.e., why they believe their proposed experiment is an original, but also meaningful, way to investigate their hypothesis using 3–5 sentences. This last assignment was especially important for us to understand why students chose this particular way of investigating the issue and how creatively and adequately they evaluate their own ideas.

### 2.5. Rating

After the transcription of all (human and AI-generated) submissions, the rating was performed by two experts from the field of psychology (two postdoctoral researchers with >12 years of experience of conducting and evaluating experimental studies), mimicking a typical review process for a grant proposal. Both raters were asked to review and evaluate responses of a scientific creativity task using a standardized rating procedure. The structured questionnaire included 13 statements (for example: *The hypothesis is adequately formulated for me to understand* or *The proposed experiment is original or creative* or *The proposed experiment is a meaningful way to test the hypothesis*). Answers to each question were given on a five-point Likert scale based on the raters’ agreement (1 = strongly disagree, 5 = strongly agree). According to this method, combining both raters’ scoring, participants could achieve a minimum score of 26 and a maximum score of 130 as an overall score. For each individual statement, the minimum score was 2 and the maximum score was 10. To ensure objective ratings unaffected by personal opinions regarding AI and its creative potential, AI-generated responses were randomly intermixed among the students’ responses. We hereby ensured that our raters were unaware that some of the responses were AI-generated. After completing the rating procedure, both raters were debriefed. The study program’s language of instruction is English and an official certificate (i.e., TOEFL iBT with minimum 80 pts or IELTS with minimum 6.0 pts) is necessary as proof of proficiency in the English language for non-native speakers. To rule out that language difficulties prevented students from expressing their ideas, we asked both raters to also judge the language proficiency for each participant.

### 2.6. Evaluation

Due to the nature of the task, we assessed both quantitative and qualitative aspects of our students’ responses. As our task is new and still being validated, we deem it necessary to discuss qualitative aspects of our results in more detail. In order to obtain a more fine-grained view, we will discuss qualitative patterns of the data and refer the reader to the supplementary data for specific text examples (see Appendix B).

For descriptive and inferential analyses, we used JASP software (JASP Team, Version 0.16.2). To classify the agreement between both raters, we calculated interclass correlations (ICC) as implemented in the JASP software. The average ICC coefficient was 0.64 (*MeanRange* = 0.423; see Table 1 as well as Appendix A for descriptive statistics) indicating a good consistency between the ratings (Cicchetti 1994).

Since the data were not normally distributed, predominantly due to limited variance in the responses generated by ChatGPT, we performed statistical analyses based on non-parametric methods (Mann–Whitney U-test). Furthermore, with limited or no variability in some response categories, not all comparisons could be analyzed quantitatively. We will therefore discuss the remaining comparisons in the following.

## 3. Results

**Figure 1:**
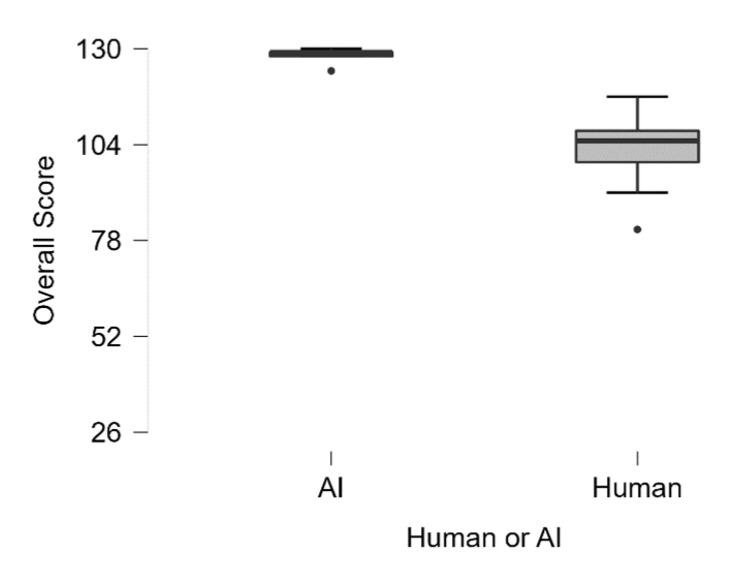
Overall sum score for scientific creativity. Participants could achieve a minimum score of 26 and a maximum score of 130.

For the overall sum score, a Mann–Whitney U-test revealed that ChatGPT received a significantly higher total rating score (Median = 129) compared to the human sample (Median = 105), U = 50, *p* < 0.003 (see Figure 1).

Analyzing the individual statements within each assignment in more detail revealed the following results:

### 3.1. Assignment 1—Generating a Hypothesis

In regard to the quality of the hypothesis, there were no significant differences between ChatGPT (Median = 10) and the human sample (Median = 9) in Q1 (Q1_The hypothesis is adequately formulated (logical) for me to understand). We further did not observe significant differences between ChatGPT (Median = 10) and the human sample (Median = 10) in Q2 (Q2_It is possible to falsify the hypothesis).

#### Qualitative Assessment

In general, students and ChatGPT performed very similar when generating a hypothesis based on the details provided in our scenario. Hence, it seems that ChatGPT is capable of formulating convincing and testable hypotheses in the opinion of both raters, very similar to the performance of our students. Also, from a descriptive point of view, although two students explicitly formulated a null as well as an alternative hypothesis, there were no obvious differences between AI and humans in terms of writing style or elaboration.

### 3.2. Assignment 2—Outlining the Procedure

**Figure 2:**
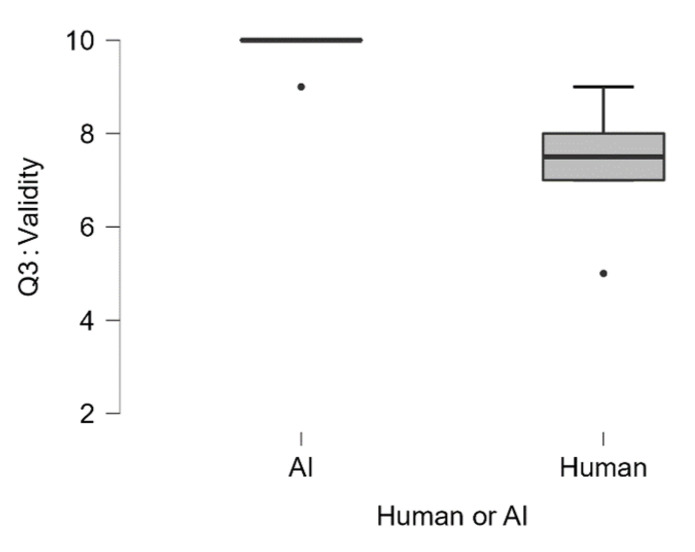
Q3: The experiment is valid. Participants could achieve a minimum score of 2 and a maximum score of 10.

Regarding the description of the experimental procedure, we observed that ChatGPT scored significantly higher (Median = 10) concerning the perceived validity of the experiment (Q3_The experiment is valid) compared to the human sample (Median = 7.5), U = 49.5, *p* < 0.002 (see Figure 2).

#### Qualitative Assessment

Both raters perceived the outlines of the experimental procedure as more valid for the AI-generated answers. However, students’ answers were more diverse in terms of the experimental methods (e.g., online surveys, food diary for several weeks, blood sampling, longitudinal vs. cross-sectional study design, investigating a special sample such as vegan or ill participants on a special diet), additional variables (e.g., consideration of cultural differences for the music taste and food preferences), as well as specific observation methods (e.g., provide participants a certain food and observe which room with two different varieties of music playing they stay in longer). Overall, students’ answers also varied in terms of the necessary steps in their experiment (between 3–5 steps), whereas ChatGPT-generated experiments always involved exactly 5 steps, always concluding with a statistical analysis as the final step). Moreover, one student added a table to show the experimental conditions of the proposed experiment.

On the other hand, ChatGPT created loose ends by initially proposing a method which was then neither used in the subsequent implementation of the experiment nor the data analysis. For example, one AI-generated procedure suggested collecting a blood sample as a baseline measurement and afterwards randomly assigning participants to a high vs. low magnesium diet for two weeks, without later using or analyzing the previously collected blood sample. In other instances, ChatGPT presented logical inconsistencies in regards to the proposed experimental procedure. For example, one AI-generated procedure included a blind taste test where participants rate the topping “without knowing the nutritional content”, which is not feasible as participants will be able to distinguish both ingredients when tasting. However, these relatively minor inconsistencies did not have an impact on the overall rating of logical flow of arguments, as both reviewers perceived the procedure as logical. This might be due to the elaborate language and overall longer explanations that ChatGPT generated to describe the experiment. It is conceivable that the sheer amount of information disguised these subtle inconsistencies.

### 3.3. Assignment 3—Listing Necessary Equipment

Due to a limitation in variance mentioned above, we were not able to perform a formal statistical analysis for Assignment 3.

#### Qualitative Assessment

When it came to listing the necessary equipment for the experiment, ChatGPT-generated lists were a lot more detailed as compared to the students and overall highly similar (e.g., using the Big Five Inventory to assess personality traits; 5×). For example, ChatGPT recommended in two proposals to collect data from 200 students, included suggestions for statistical software packages (4×), and also indicated to provide consent forms and information materials (1×). Overall, the equipment listed by ChatGPT was more detailed, whereas students did not list material such as, for example, the statistical software they want to use for analyzing the data. This again shows that ChatGPT is capable of providing very specific details that humans could possibly forget as they might, for example, take it for granted to have statistical software available. In this instance, ChatGPT definitely profits from its LLM structure and its comprehensive database consisting predominantly of entries that have been successfully rated and thus funded in reality; hence, only this positive selection became the basis of recent scientific studies and manuscripts. This training set will likely help to list extensive materials, especially if the AI training data also includes actual scientific reports or even just popular science articles. Here, it is arguable that using AI to assist in compiling necessary material could indeed be beneficial for writing research proposals.

### 3.4. Assignment 4—Reasoning of Rationale

**Figure 3:**
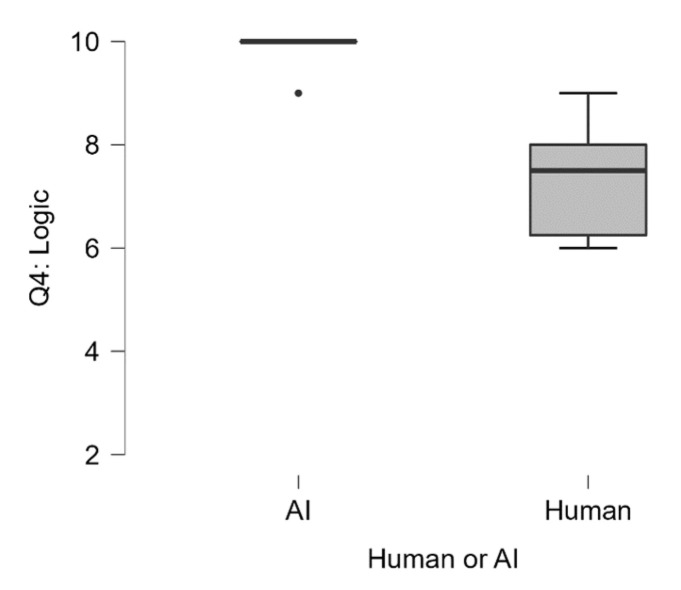
Q4: The student’s rationale is logical. Participants could achieve a minimum score of 2 and a maximum score of 10.

In respect to the logical outline of the experiment (Q4_The student’s rationale is logical), we observed that ChatGPT scored significantly higher (Median = 10) as compared to the human sample (Median = 7.5), U = 49, *p* < 0.003 (see Figure 3). However, we did not observe significant differences between ChatGPT (Median = 10) and the human sample (Median = 8.5) in terms of how adequate the proposed experiment is perceived by the two raters (Q5_The student’s rationale is adequately formulated for me to understand).

**Figure 4:**
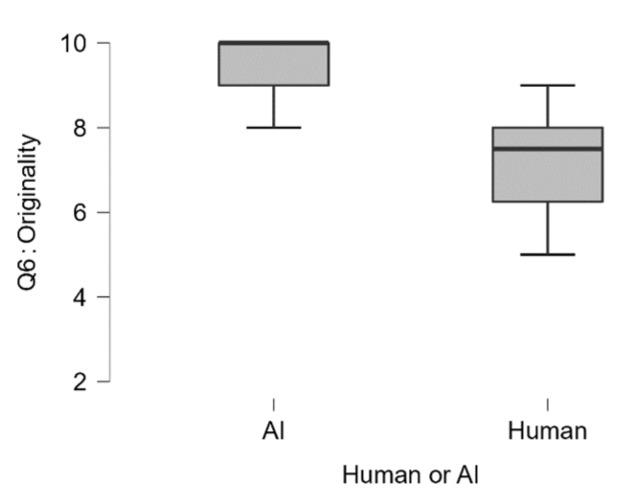
Q6: The proposed experiment is original or creative. Participants could achieve a minimum score of 2 and a maximum score of 10.

**Figure 5:**
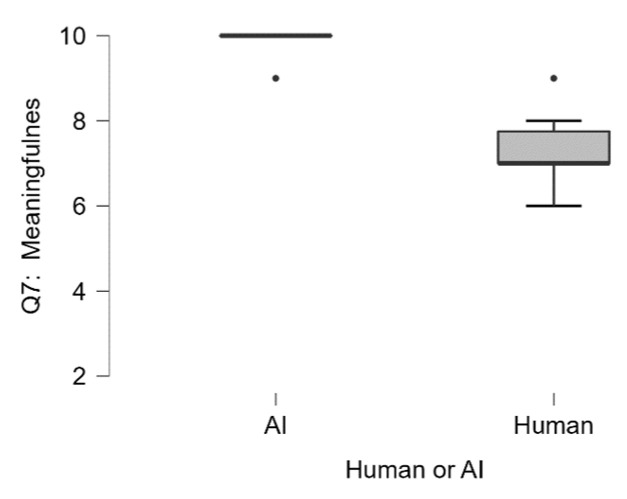
Q7: The proposed experiment is a meaningful way to test the hypothesis. Participants could achieve a minimum score of 2 and a maximum score of 10.

In regards to how original or creative the raters find the proposed experiment (Q6_The proposed experiment is original or creative), we observed that ChatGPT scored significantly higher (Median = 10) as compared to the human sample (Median = 7.5), U = 46.5, *p* < 0.008 (see Figure 4). We obtained very similar results in terms of meaningfulness (Q7_The proposed experiment is a meaningful way to test the hypothesis), where ChatGPT scored significantly higher (Median = 10) as compared to the human sample (Median = 7), U = 49.5, *p* < 0.002 (see Figure 5).

#### Qualitative Assessment

With regards to justifying the rationale on why the proposed experiment is an original, but also meaningful, way to investigate the hypothesis, both raters perceived the logical outline of ChatGPT as more convincing in contrast to the students. Similarly, both experts rated ChatGPT-generated rationales as more original and they perceived them as a more meaningful way to test the hypothesis, in contrast to the human sample. By analyzing the content of those answers, it is striking that ChatGPT argues on a more conceptual, comprehensive level on why the experiment is meaningful (i.e., because it combines personality psychology with nutritional science). On the other hand, students argued on an operational level (e.g., Student A: determination of a possible effect of magnesium on musical preferences; Student B: isolating dietary factors from personality traits to determine their relative influence on music preferences; Student C: repeating the experiments with people from different cultural backgrounds). This difference in conceptual level of argumentation could be an indicator on how to better train students and young scientists on how to formulate more convincing research proposals.

Lastly, our participants’ language proficiency was rated. On average, students achieved a score of 4.67 out of 5 (*SD* = 0.64), which indicated that there was no problem for the raters to understand participants’ ideas.

To summarize, ChatGPT outperformed our students, not only in regards to the overall score, but crucially also in critical sub-scores, such as originality or meaningfulness. However, our additional qualitative analysis revealed more subtle differences when comparing the answers of ChatGPT in contrast to the students.

## 4. Discussion

With this new task, we attempt to shed light on whether ChatGPT can match or outperform graduate cognitive science students in their scientific creativity, measured by writing a short research proposal based on four specific assignments (i.e., formulating a hypothesis, designing an experiment and listing the required material to test the hypotheses, and justification on the chosen method).

Very similar to the study of Vinchon et al. (2024), we did not observe much variety in the proposals generated by ChatGPT, especially in terms of the outline of the experimental procedure as well as the justification of the experiment’s rationale (see above). The fact that AI-generated ideas are so extremely similar and have minimal variance, while human creativity is assumed to be normally distributed, can be explained with the architecture of an LLM. Generating texts by selecting each word because it has a certain statistical probability of following its predecessor necessarily produces many similarities. At the same time, it underlines that AI-generated creativity is not comparable to human creativity in generating independent ideas of various qualities but rather on repeating what is presumed to be the best idea based on its high probability (Farina et al. 2024; Lockhart 2024). This in turn would reduce the very definition of high originality (i.e., statistical rarity) to absurdity.

Another explanation could be that, due to the wording in our scenario, the alternative hypothesis could already be inferred. The precise wording of prompts is important as it strongly influences the answers generated by ChatGPT in other creativity tasks (Guzik et al. 2023). As we aimed to keep the ideas generated by students comparable, the scenario was very closely defined. As this might be too obvious for humans to cause any inferences, at the same time this might have given an advantage to ChatGPT as it could use this information to generate its answers as well. Notably, in our study, the first step of the scientific process—phrasing the research question—is already provided to allow for a valid comparison of the responses. Future research in new formats is needed to address the issue of how these initial questions are formed by both humans and AI.

At the same time, we can only hypothesize about the impact that plagiarism has on the answers generated by ChatGPT. For example, in the aforementioned study, ChatGPT delivered several noticeably plagiarized texts when prompted to invent an original story (Vinchon et al. 2024). Furthermore, the authors discussed that these generated responses may look creative, but are actually only plagiarism of a (very) creative (human) idea. In our study, we can only hypothesize how much ChatGPT could potentially have used plagiarism to solve our task due to its large (and for the general public not disclosed) dataset. A prompt in Google Scholar including the words NUTRITION and PERSONALITY and INTROVERT and EXTROVERT returned more than 6000 entries (as of February 2025). Hence, chances are that ChatGPT has used the previous ideas of other scientists to shuffle its answers. This also shows that ChatGPT hallucinated its justifications on why the proposed experiment is original (i.e., because it combines personality psychology with nutritional science, which has obviously already been achieved), and shows the disadvantage that ChatGPT does not “understand” what it is doing and does not protect intellectual ownership (Sternberg 2024). Hence, the answers given by our students that are all on a more operational level could indeed be creative or at least an interesting addition to the “existing” data in our fictious scenario (e.g., repeating the experiments with individuals from different cultural backgrounds). To investigate this issue, in the future we could vary the task and provide students access to the internet (such as Google Scholar or PubMed) when solving our task. This could make the comparison a little fairer. Another approach would be to provide students access to ChatGPT, so we could compare AI–human co-creativity, which is beginning to become the new reality even in academia. Our study can be understood as an initial step towards assessing scientific creativity precisely in humans vs. generative AI separately. This could help to identify productive ways how generative AI can assist researchers at different steps along the scientific process. It remains to be seen whether the impact of generative AI will differ across different scientific disciplines. We argue that the benefit of using AI largely depends on the specificity of each problem, very similar to how human creativity requires different cognitive processes in response to open or closed problem spaces (i.e., divergent and convergent thinking; see (Eymann et al. 2024b) as well as (Jaarsveld and Lachmann 2017) for overviews).

Regarding the qualification of research ideas, our observations show that we face a few shortcomings when it comes to the assessment of scientific creativity. In our study, we used a structured rating procedure with two experts from the specific field. While this is a suitable way to qualify human creativity, it could be more difficult to assess AI-generated creativity. There are several studies that have found specific effects of human creativity ratings. For example, Licuanan et al. (2007) reported that raters underestimated the originality when it comes to extremely creative in contrast to average creative ideas. The authors further concluded that raters had problems with distinguishing popular from truly original ideas because this information is not readily accessible (Licuanan et al. 2007). Sternberg (2024) evaluated this problem, stating that a small creative improvement is oftentimes easier to comprehend and interpret than outstanding creativity. And this could be even true for experts in a respective scientific field, who do not always agree. Furthermore, history has shown that outstanding creativity will oftentimes be recognized with time, years or sometimes only centuries later, even by contemporary experts.

Another problem arises when specifically investigating AI-generated ideas. For example, Si et al. (2024) found that LLM-generated research ideas were rated significantly higher in terms of novelty by human raters as opposed to human expert ideas, which may also have played a role in our study. Si et al. (2024) concluded that the judgement of novelty and originality might be difficult to rate even for experts and they further propose to evaluate entire research projects, rather than exclusively research ideas. While our study is a first attempt to assess this idea, the task could be improved by also adding specific value-based decisions such as ethical approvals or social responsibilities to our task. A key element of scientific progress is not only innovation; scientists also need to make ethical or moral decisions, especially when investigating human participants. This is, until today, a fundamental limitation of AI (Opara 2025). Furthermore, a more fine-tuned textual analysis (even performed by AI) could be beneficial to determine some of the more subtle differences between human and AI-generated proposals, and with that account for the shortcomings that a rating procedure faces.

## 5. Conclusions

As AI enters the field of creativity, it is necessary to recalibrate our current understanding of creativity (Runco 2023). This is not only true for the AI artistry (Garcia 2024), but potentially for scientific creativity as well. In this regard, it might be appropriate to move the focus from scoring ideas in terms of fluency, flexibility, and originality to other focal points.

Instead, it could be beneficial to evaluate any scientific output (such as hypothesis, theory, model, or methodology) in terms of their “pursuitworthiness” (Sánchez-Dorado 2023, p. 81), which describes the potential to provide a specific epistemic benefit (Sánchez-Dorado 2023). Although several outstanding scientists have emphasized making unobvious associations and using intuition when thinking creatively about science, in the end in science it is not about making ideas as extraordinary as possible but “suitable” or “appropriate” for the scientific problem. This could put the emphasis away from categories that can easily be outperformed by AI (e.g., fluency, flexibility, or even originality, see (Guzik et al. 2023)) onto features that are harder to simply infer from pre-existing databases and more targeted towards proper (human) creativity, vision, and even intuition (Lubart et al. 2022).

Similar to Sternberg (2024, p. 4), who asked “And now, with generative AI, why learn how to write a paper when one of the generative AI programs can write it for you?”, we could also pose the question whether it makes sense to teach students how to write a proposal, when AI can seemingly also do it, and even more convincingly. We argue for shifting our view on scientific creativity away from focusing only on traditional creativity measures onto the question of whether these scientific problems “are even worth solving” (Sternberg 2024, p. 6). With that, AI could assist scientists rather than replace human creativity in scientific environments.

## Figures and Tables

**Table 1:** ICC correlations and range for each item indicating the degree of agreement of both raters. Note: R1 = Rater 1; R2 = Rater 2.

*Variable*	*Q1 Adequate*	*Q2 Falsification*	*Q3 Validity*	*Q4 Logic*	*Q5 Adequate*	*Q6 Originality*	*Q7 Meaningfulness*
***ICC***	0.606	0.876	0.684	0.453	0.56	0.7	0.621
***Range R1***	2.0	4.0	4.0	3.0	3.0	4.0	3.0
***Range R2***	4.0	4.0	1.0	2.0	1.0	2.0	1.0

## Data Availability

The data presented in this study are available on request from the corresponding author. The data are not publicly available due to privacy reasons.

## Abbreviations

The following abbreviations are used in this manuscript:

AI	Artificial Intelligence
GPT	Generative Pre-trained Transformer
LLM	Large Language Model

## Appendix A

**Table A1:** Descriptive statistics for each item. Please note that Q5 Adequate was not formally tested due to limited variance.

	Overall Score		Q1 Adequat		Q2 Falsification		Q3 Validity		Q4 Logic		Q5 Adequat		Q6 Originality		Q7 Meaningfulness	
	AI	Human	AI	Human	AI	Human	AI	Human	AI	Human	AI	Human	AI	Human	AI	Human
Median	129.00	105.00	10.00	9.00	10.00	10.00	10.00	7.50	10.00	7.50	10.00	8.50	10.00	7.50	10.00	7.00
Mean	128.00	102.10	9.80	8.40	9.60	8.30	9.80	7.40	9.80	7.40	9.80	8.30	9.40	7.10	9.80	7.20
Std. Deviation	2.35	10.09	0.45	2.17	0.55	2.91	0.45	1.07	0.45	1.17	0.45	1.57	0.89	1.37	0.45	0.92
Range	6.00	36.00	1.00	6.00	1.00	8.00	1.00	4.00	1.00	3.00	1.00	4.00	2.00	4.00	1.00	3.00
Minimum	124.00	81.00	9.00	4.00	9.00	2.00	9.00	5.00	9.00	6.00	9.00	6.00	8.00	5.00	9.00	6.00
Maximum	130.00	117.00	10.00	10.00	10.00	10.00	10.00	9.00	10.00	9.00	10.00	10.00	10.00	9.00	10.00	9.00

## Appendix B

*Sample answers for each assignment of the task.*

**Assignment 1. Generating a hypothesis**

A. Example Student:

*Preference for music does not depend on a person’s diet.*

B. Example ChatGPT:

*Music preference (rock* vs. *chill-out) among introverted and extroverted university students is primarily influenced by personality traits rather than dietary factors, such as magnesium content in food.*

**Assignment 2. Outlining the procedure**

A. Example Student:

*Introvert students are recruited for the experiment. Their musical preferences are recorded. Then they will be randomly split into 3 groups:*

*1. eggplant-supplementation (they must eat eggplant daily)*

*2. magnesium-supplementation (in pill form, the daily recommended dosage, taken each day)*

*3. No intervention*

*The experiment will last 4 weeks. After 4 weeks, the participants are invited back to the lab and fill out a questionnaire on their musical preferences (same as at the start). Additionally, the same number of extroverts are recruited and also distributed in 3 groups to allow for a control condition. Magnesium levels are determined at start and end for each student.*

B. Example ChatGPT:

*1. Recruitment and Grouping: Recruit 200 university students (equal numbers of self-identified introverts and extroverts). Use a validated personality inventory (e.g., the Big Five Inventory) to confirm their personality traits.*

*2. Baseline Measures: Assess participants’ current dietary habits, magnesium levels (via a simple blood test), and pre-existing music preferences through a standardized questionnaire.*

*3. Dietary Intervention: Randomly assign participants within each personality group to either a high-magnesium diet (including eggplant and other magnesium-rich foods) or a control diet for 2 weeks.*

*4. Music Preference Testing: After the dietary intervention, use a blind-listening test where participants rate their preference for various music genres (rock, chill-out, pop, classical, etc.).*

*5. Analysis: Compare music preferences within and across personality and dietary intervention groups using statistical methods (e.g., ANOVA).*

**Assignment 3. Listing necessary equipment**

A. Example Student:

*- Questionnaires (music enjoyment, flavor enjoyment, intro-/extroversion*

*- Speaker for playing music (and computer for selecting music)*

*- Pizza*

B. Example ChatGPT:

*- Validated personality inventory (e.g., Big Five Inventory)*

*- Pizza with customizable toppings (peppers, mushrooms, eggplant, corn, etc.)*

*- Music playback system with a standardized playlist (rock and chill-out genres)*

*- Survey tools or software to record music preferences (e.g., Likert scales, ranking systems)*

*- Statistical software for data analysis (e.g., SPSS, R)*

**Assignment 4. Reasoning of rationale**

A. Example Student:

*This study allows for the participants to freely choose their music after eating each type of pizza, which means there should be less confounds. The food diary also helps see if participants have eaten more magnesium containing food over the past couple of weeks that could influence them. By taking personality into account this also allows for more comprehensible results.*

B. Example ChatGPT:

*This experiment is original because it combines an interdisciplinary approach, linking psychology, nutrition, and music preferences, to examine the underlying drivers of seemingly correlated behaviors. By disentangling personality traits from dietary influences, the study addresses a novel intersection of psychological and physiological factors. The results could advance our understanding of how personality shapes preferences and behaviors, contributing to fields such as consumer psychology and dietary-behavioral research. If successful, the study could challenge simplistic dietary explanations of behavior, highlighting the complex interplay between personality and external choices.*
